# The impact of contact with nature on positive youth development: a multiple mediation model

**DOI:** 10.3389/fpsyg.2025.1514244

**Published:** 2025-04-08

**Authors:** Yulan Li, Liling Mo, Caiyan Tan, Jin Fu, Niyan Wang

**Affiliations:** ^1^School of Education Science, Nanning Normal University, Nanning, China; ^2^Center for Applied Mathematics of Guangxi, School of Mathematics and Statistics, Nanning Normal University, Nanning, China; ^3^School of Education, South China Normal University, Guangzhou, China; ^4^Harrow Lide School Nanning, Nanning, China

**Keywords:** contact with nature, positive youth development, biophilia hypothesis, stress recovery theory, attention restoration theory

## Abstract

**Introduction:**

In recent years, there has been increasing evidence supporting the benefits of contact with nature. Little is known about the mechanisms of how nature contact lead to these benefits. Positive youth development refers to the state in which adolescents strive to achieve full, healthy, and successful growth, which has been an important goal for societies. Against this background, the present study aimed to examine whether and how adolescents’ contact with nature influences positive youth development in a sample of Chinese adolescents.

**Methods:**

Drawing on the biophilia hypothesis, stress recovery theory, and attention restoration theory, 1,730 junior high school students participated in a questionnaire survey. The study employed a multiple mediation model to explore the effects of adolescents’ contact with nature on positive youth development, mediated through mindfulness, connectedness to nature, and perceived stress.

**Results:**

Adolescents’ contact with nature positively predicted the level of positive youth development, while contact with artificial environments did not. Contact with nature not only directly predicted the level of positive youth development but also indirectly influenced it through the mediating effects of connectedness to nature and perceived stress, but the mediated effect of mindfulness was no significant.

**Discussion:**

These findings provide empirical support for the biophilia hypothesis and stress recovery theory. They also have important implications for urban planning and school policies, suggesting that ensuring adolescents have access to and opportunities to engage with natural environments is crucial for promoting positive youth development.

## Introduction

Adolescents are human capital for the future and promoting their positive and healthy growth is an important strategy goal in all countries around the world. However, in the field of child development, traditional research has often focused on adolescents’ mental and behavioral problems from a prevention and reduction perspective. Researchers and practitioners have looked for and intervened with problems or risk factors that may exist in adolescents. In fact, such problem-focused solutions often do not work as expected, high-risk behaviors in adolescents are still in high incidence even that an intervention is implemented (Carnegie Council on Adolescent Development, 1995). This “deficit-prevention” model has been criticized by proponents of positive psychology. Emerging in the 1990s, the Positive Youth Development (PYD) perspective emphasizes the importance of fostering adolescents’ strengths and potential, promoting their overall healthy and successful development, rather than merely reducing problematic behaviors (Damon, 2004). To promote adolescents’ PYD, the theories in this field such as Developmental Contextualism, Relational Developmental Systems, and Developmental Assets emphasize the individual-environment interaction. It is evident that encouraging adolescents to engage in community activities can increase their connection with the community, and improve their level of PYD and reduce problematic behaviors (Edberg et al., 2017). Likewise, Nature serves as a valuable external ecological asset for fostering healthy and successful development in adolescents has gradually received attention (Bowers et al., 2021).

Throughout human evolution, people have lived closely connected to the natural environment, making contact with nature an essential part of life. However, with the rapid advancement of industrialization and urbanization, the majority of humans now live in cities, distanced from nature. Evidence suggests that adolescents today spend significantly less time outdoors compared to previous generations (Larson et al., 2019). In his book *Last Child in the Woods: Saving Our Children from Nature-Deficit Disorder*, Louv (2008) argues that children and adolescents growing up in modern cities are increasingly isolated from nature, leading to what he terms “nature-deficit disorder,” which negatively impacts their physical and mental health. In recent years, the benefits of nature contact have become a focal point of research in psychology and environmental science. Nature contact refers to the interaction between human beings and the natural environment and encompasses related concepts such as natural exposure and nature experience. It includes both direct contact (e.g., outdoor activities like playing in the park or walking in the woods) and indirect contact (e.g., watching pictures or videos featuring natural elements) (Hartig et al., 2014). A significant body of research has shown that contact with nature can enhance individuals’ physical and mental health, cognitive function, academic performance (Bratman et al., 2012; Chen et al., 2016). It has also been found to impact PYD positively among adolescents in low-income communities in the United States (Bates et al., 2018; Bowers et al., 2021). However, the mechanisms through which nature contact leads to these benefits remain largely unclear (Kondo et al., 2020). Further empirical studies are needed to explore how nature contact contributes to positive health outcomes and to better understand the processes underlying these effects. In addition, the cultural factor might influence individuals’ perception of nature and understanding of PYD, and most studies about nature contact and child development have been conducted in Western cultural contexts. In the Chinese cultural context, there is a strong emphasis on the harmony between humans and nature, with values such as “keeping nature balanced” and “living in harmony with nature.” Whether contact with nature has similar positive effects on PYD in Chinese adolescents has yet to be thoroughly examined.

### The Chinese 4Cs model of positive youth development

The structure of PYD is a core issue concerned for researchers in this realm. After reviewing 25 adolescent development projects in the United States, Catalano et al. (2004) firstly proposed 15 goals for PYD, including resilience, connectivity, connection, competence, positive self-identity, prosocial behavior and so on. Lerner et al. (2005) suggested that adolescents can thrive when their personal strengths were combined with key environmental resources in their lives, encapsulated in the 5Cs Model, which includes competence, character, confidence, connection, and caring. Currently, the PYD measurement based on the 5Cs model is widely used internationally (Geldhof et al., 2014; Johnson and Ettekal, 2023; Robinson et al., 2016). Whereas, the 5Cs model is evidenced by some studies to have a poor psychometric profile among adolescents in other cultures (e.g., China) (Chen et al., 2018; Conway et al., 2015). Lerner et al. (2019) also noted that any one Western-developed PYD model may omit or underestimate some potentially unique culture factors related to child development in other cultures. For example, interpersonal relationships are more important in Chinese culture, while self-worth is more important in the western culture (Shek and Ma, 2010); and the Chinese have been deeply influenced by Confucianism and Taoism; cultural difference and the other sociocultural factors may influence Chinese perceptions of and the construction of PYD. In this case, Chinese researchers proposed a four-factor model of PYD (i.e., competence, confidence, character, and connection; 4Cs model) through an integrative etic-emic approach, to align culture-specific (emic) and cultural-general (etic) aspects of PYD (Chai et al., 2022; Lin et al., 2017).

- Character refers to the skills, behaviors, and attributes that adolescents need to develop to become capable and well-adjusted within their social and cultural context, including Benevolence (Ai, 爱, e.g., kindness, filial piety and patriotism); Determination (Zhi, 志, e.g., diligence and lofty aspirations); Trustworthiness (Xin, 信, e.g., honesty and self-discipline); and Perseverance (Yi, 毅, e.g., persistence) which are indexed four Confucian values.
- Competence pertains to adolescents’ perception of their abilities and potential in various areas, including academic, social–emotional, and living competence.
- Confidence, or self-worth, refers to adolescents’ perception, emotional experiences, and behavioral tendencies regarding their own value, including positive self-concept and self-acceptance.
- Connection involves adolescents’ awareness of and interactions with the social relationships around them, including positive bonds with family, school, and community.

The model for Chinese PYD is largely retained four Cs of the 5Cs model of PYD. However, it’s worth noting that the C of character in the Chinese PYD model is the most culture-specific component. Meanwhile, compare to the C of competence, the Chinese version of competence includes living competence instead of physical competence, which also reflects the societal realities that Chinese people value the cultivation of children’s independent living ability. The C of caring is absent in Chinese 4Cs model because the overlap between caring and other indicators, such as benevolence (a component of character) and connection in Chinese culture. In a word, the 4Cs model is contextualized in the contemporary cultural and societal conditions in mainland China, and at best a close approximate of actual PYD processes among contemporary Chinese youth (Chai et al., 2022).

### The effect of contact with nature on individuals’ positive youth development

As noted above, the relationship between contact with nature and PYD has been well-documented in Western populations (Bates et al., 2018; Bowers et al., 2021). It has been observed that adolescents who frequently engage with nature become more aware of their impact on the environment and are more likely to take responsibility for its protection (Jaiswal and Bihari, 2020), contributing to the development of their “character.” Similarly, participating in activities in natural settings can strengthen adolescents’ sense of belonging and attachment to both the environment and their community (Flanagan et al., 2019; Larson et al., 2018), encouraging them to engage in community activities and thereby fostering their “connection.” Moreover, engaging in nature-based activities (such as camping) can help develop adolescents’ motor skills and life skills (Flanagan et al., 2019), enhancing their sense of competence (ability) and self-concept (self-confidence). Based on this evidence, Hypothesis 1 of the present study posits that compared to contact with artificial environment, contact with nature will positively predict PYD among Chinese adolescents.

### The theoretical framework of contact with nature

The Attention Restoration Theory, the Biophilia Hypothesis, and the Stress Recovery Theory are commonly used to explain the benefits of contact with nature (Chen et al., 2016). According to Attention Restoration Theory, frequent exposure to nature (such as trees, grass, gardens, etc.) can reduce ego depletion and energy loss caused by sustained directed attention, thereby helping individuals recover from mental fatigue (Kaplan and Kaplan, 1989). Empirical studies have shown that individuals living in environments with natural landscapes (e.g., trees, flowers, and plants) perform significantly better on attention-related tasks than those living in environments without such landscapes (Tennessen and Cimprich, 1995). Likewise, adolescents who have more green plants visible from their home windows have been found to perform better on attention tests, impulse inhibition tests, and delayed gratification tests—all of which are related to positive future development (Taylor et al., 2002).

Nature contact helps restore attention, enabling individuals to focus more effectively on a particular object or activity while disregarding external distractions. This improved ability to sustain attention (also known as mindfulness) which allows individuals to fully engage in their current activities and maintain focus over extended periods (Higgins and Turnure, 1984). Studies have found a positive correlation between an individual’s mindfulness and their efficiency, organizational citizenship behaviors, and overall performance (Nourafkan et al., 2023), and organizational citizenship behaviors is considered as a manifestation of character. Individuals with higher levels of mindfulness can allocate more energy to tasks, become more deeply immersed in their work, and achieve better results, leading to greater happiness and achievement (Niu and Huang, 2019). Based on these findings, Hypothesis 2 of the present study suggests that mindfulness partly mediates the relationship between contact with nature and Chinese adolescents’ PYD.

According to the Biophilia Hypothesis of nature contact, humans are inherently inclined to connect with nature and other living beings, not only on a physical level but also on a psychological one. The more individuals engage with nature, the more they can reconnect with their evolutionary roots, leading to improved health and happiness (Wilson, 1986). Connectedness to nature refers to the extent to which people feel emotionally and experientially linked to nature and consider themselves a part of it. Some researchers suggest that this connectedness is a crucial psychological mechanism through which nature influences human well-being, and it is a significant variable in the human-nature relationship (Zelenski et al., 2015). Nature contact enhances this connection, and the more frequently an individual contacts with natural environment, the stronger their sense of connectedness to nature, which in turn contributes to better personal development (Cheng and Monroe, 2012). Therefore, this study proposes Hypothesis 3: connectedness to nature mediates the relationship between contact with nature and Chinese adolescents’ PYD.

According to Stress Recovery Theory, being in nature helps reduce stress by lowering the sensitivity of the nervous system to stressors. Perceived stress refers to an individual’s psychological response to stress after cognitive evaluation, often manifested as tension, discomfort, and a sense of being overwhelmed or out of control (Ulrich, 1983). Perceived stress is a predictor of anxiety, and individuals with higher levels of perceived stress are more likely to adopt passive coping strategies (Feng et al., 2023). Research has shown that individuals who walk in natural environments experience significant perceptual recovery and reduced amygdala activity, which helps prevent anxiety (Sudimac et al., 2022). Children and adolescents who report greater exposure to nature in their home and school environments—such as campus greenery, community green spaces, and natural landscapes visible from windows—tend to have lower levels of perceived stress compared to those with less exposure (Corraliza et al., 2012). Based on these findings, this study proposes Hypothesis 4: perceived stress mediates the relationship between contact with nature and Chinese adolescents’ PYD.

### The present study

In summary, the relationship between natural contact and adolescents’ PYD has been well-documented in Western samples, however, the mechanism of how contact with nature leads to PYD remains unclear. Due to cultural differences, Chinese people have different perception on nature and understanding of PYD, very little is currently known about whether contact with nature leads to Chinese adolescents’ PYD. Based on the Chinese 4Cs model of PYD, the present study aims to explore whether and how contact with nature influences PYD among Chinese middle school students. According to literature reviews and three theories (i.e., Attention Restoration Theory, Biophilia Hypothesis, and Stress Recovery Theory), this study proposes 4 hypotheses as follows.

H1: compared to contact with artificial environment, contact with nature will positively predict PYD among Chinese adolescents.

H2: mindfulness partly mediates the relationship between contact with nature and Chinese adolescents’ PYD.

H3: connectedness to nature mediates the relationship between contact with nature and Chinese adolescents’ PYD.

H4: perceived stress mediates the relationship between contact with nature contact and Chinese adolescents’ PYD.

**Figure 1:**
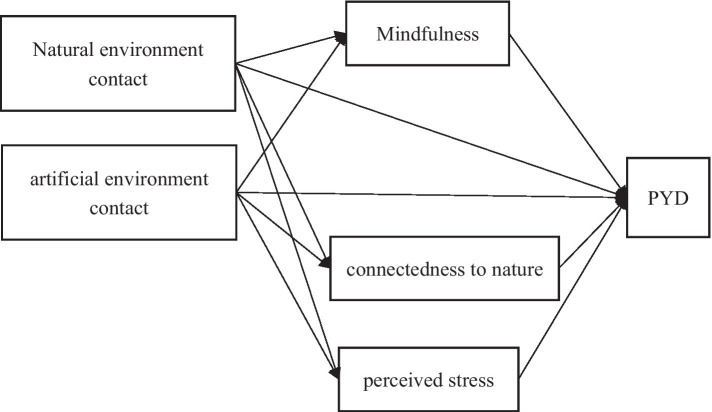
The Hypothetical multiple mediated model.

Based on the hypotheses, a multiple mediation model is constructed and illustrated Figure 1.

## Methodology

### Participants

A total of 1,827 students in Grades 7–9 from 10 middle schools in Nanning, Liuzhou and Guilin, Guangxi—a province in southern China—participated in this study. All participants were provided with parental consent forms, which needed to be signed and returned to their headteachers for participation in the survey. Questionnaires with more than 10% missing data and the questionnaires which did not report the basic demographic information (i.e., gender and age) were excluded from the analysis. Consequently, 1,730 students were included in the final sample, resulting in a valid response rate of 94.69%. The sample included 473 students in Grade 7 (50.7% female, M_age_ = 12.67, SD = 0.55), 997 students in Grade 8 (53.9% female, M_age_ = 13.58, SD = 0.51), and 260 students in Grade 9 (48.1% female, M_age_ = 14.62, SD = 0.57).

### Measurements

#### Positive youth development

The Chinese Positive Youth Development Scale (short version) developed by Chai et al. (2022) was used to assess the level of PYD. This scale was a 48-item self-reported measure comprising four subscales: character, competence, confidence, and connection. Example items were “when I see someone in need, I’ll do my best to help him” for character, “I can do housework well” for competence, “I know many positive qualities or strengths about myself” for confidence, and “In addition to my family, there are people in my neighborhood who really care about me” for connection. Each item was rated on a 5-point Likert scale, ranging from 1 (strongly disagree) to 5 (strongly agree). Higher scores indicated a higher level of PYD. The internal consistency coefficients (Cronbach’s *α*) for the subscales were as follows: character (*α* = 0.96), competence (*α* = 0.93), confidence (*α* = 0.95), and connection (*α* = 0.94). The overall internal consistency of the scale was excellent (*α* = 0.98).

#### Contact with natural and artificial environment

According to Preuß et al. (2019) opinions, natural environments were the public and private open spaces that contain green and/or blue natural elements, including forests, farmland, city parks, roof gardens, canals, ponds rivers, lakes and beaches. Considering preventing adolescent drowning was an important work in Chinese schools, we excluded the “blue” elements in the survey. Based on the investigation of nature exposure time (Beyer et al., 2016; Van den Berg et al., 2016), participants were asked about the frequency and average duration of time spent in natural environments (e.g., parks, farmland, gardens and orchards) in a typical daytime (7:00–18:00) and night (after 18:00) over the past month (4 weeks), because many Guangxi people preferred go out for activities at night due to hot weather, and students had studied in school during the daytime, and their leisure time was only at night. Correspondingly, the questions also developed to ask about the frequency and average duration of time spent in artificial environments (e.g., city streets, playgrounds, city squares). In this case, there were 8 items in this instrument. The response options for contact frequency were: Never, Once, 2–3 times/month, 1–2 times/week, 3–4 times/week, and Almost every day, scored from 1 to 6, respectively. The response options for the average duration of each contact were: Less than 1 h, 1–2 h, 2–3 h, 3–5 h, and More than 5 h, scored from 1 to 5, respectively. The frequency and average duration scores were multiplied to create indices for natural and artificial environment contact. Confirmatory factor analysis indicated that the two-factor model of natural/artificial environment contact was well-fit (χ^2^/df = 2.58, GFI =0.99, NFI = 0.99, TLI = 0.99, CFI = 0.99, RMSEA = 0.03). The internal consistency of the scales for natural and artificial environment contact were *α* = 0.83 and *α* = 0.84, respectively.

#### Mindfulness

A 6-item scale was used to assess the degree to which an individual was fully focused on the task at hand. The scale was adapted by Chinese scholars based on the Kentucky Inventory of Mindfulness Skills and the Mindfulness Attention Awareness Scale (Niu and Huang, 2019). An example item was “I’m worried about something or distracted so that I do not notice what I’m doing (Reversed).” Each item was rated on a 5-point Likert scale, ranging from 1 (strongly disagree) to 5 (strongly agree). Higher scores indicated a higher level of mindfulness. The internal consistency of the scale was good, with a Cronbach’s *α* of 0.89.

#### Connectedness to nature

The Chinese version of the Connectedness to Nature Scale (CNS), developed by Mayer and Frantz (2004), was used to measure the emotional connection between individuals and the natural world (Li and Wu, 2016). This one-dimensional scale consisted of 14 items, each describing aspects of the emotional bond with nature. An example item was “I often feel at one with nature.” Responses were rated on a 5-point Likert scale, ranging from 1 (strongly disagree) to 5 (strongly agree). Higher scores indicated a stronger emotional connection to nature. The internal consistency of the scale was excellent, with a Cronbach’s α of 0.91.

#### Perceived stress scale

The Chinese version of the Perceived Stress Scale, developed by Cohen et al. (1983) was used to assess the level of perceived psychological stress (Yang and Huang, 2003). The scale consisted of 14 items divided into two dimensions: tension and loss of control. An example items was “I feel nervous and stressed.” Each item was rated on a 5-point Likert scale, ranging from 1 (strongly disagree) to 5 (strongly agree). Higher scores indicated greater perceived psychological stress. The internal consistency of the scale was good, with a Cronbach’s α of 0.88.

### Procedural framework

Using convenience sampling, the researchers contacted schools to request their participation in the study. A total of 10 schools, located in both urban and rural areas of Nanning, Liuzhou, and Guilin—the top three cities in Guangxi—agreed to take part. There was little difference among these 10 schools in terms of school buildings, sports venues, teaching facilities and equipment due to the policy of “the Balanced Development of Compulsory Education” which was implemented in 2012. This policy aimed to promote the standardization of schools in which all children and adolescents can receive national-standard compulsory education (Ministry of Education of the People's Republic of China, 2012). According to the Ministry of Education annual monitoring report of 2,764 districts, the comprehensive coefficient of variation of junior middle schools was 0.322, which were in a good level (Ministry of Education of the People's Republic of China, 2020). Meanwhile, these 10 schools were in the districts which had passed the national assessment of the basic balanced development of compulsory education (Department of Education of Guangxi Zhuang Autonomous Region, 2024).

Data was collected in the spring semester of 2023 (May and July). Parental consent forms were distributed to students via their teachers the day before the survey was conducted. During one class period on the next day, a trained graduate student majoring in psychology acted as a research assistant, distributing the paper-questionnaire packets to the students in a classroom setting. The students were required to report their gender and age, and then completed the questionnaires anonymously and independently, taking approximately 20 min to finish. As a token of appreciation, participants received a small gift, such as a pen, notebook, or pencil case, for their participation.

## Results

### Preliminary analyses

Since all the measures in the present study were self-reported, the Harman single-factor test was used to check for common method bias. The results showed that 20 factors had eigenvalues greater than 1, and the first factor accounted for 28.29% of the variance, which is below the critical threshold of 40% (Podsakoff et al., 2003). This suggests that common method bias is not a significant issue in this study.

### Descriptive statistics and correlation analysis

Gender and grade were dummy coded, and means, standard deviations, and intercorrelations of all variables were calculated. As shown in Table 1, gender was negatively and significantly correlated with natural environment contact, artificial environment contact, and three dimensions of PYD (excluding character), as well as positively and significantly correlated with mindfulness and perceived stress. This indicated that boys scored higher than girls in natural environment contact, artificial environment contact, and three dimensions of PYD, while boys scored lower than girls in mindfulness and perceived stress.

**Table 1:** Descriptive statistics and correlation analysis for all variables.

	Gender	Grade 8	Grade9	X1	X2	M1	M2	M3	Character	Competence	Confidence	Connection
Gender	1																					
Grade 8	0.04	1																			
Grade 9	−0.03	−0.49**	1																	
X1	−0.00**	0.06*	−0.02	1															
X2	−0.13**	−0.03	0.03	0.66**	1													
M1	0.05*	0.03	−0.06*	−0.05*	−0.12**	1											
M2	−0.06*	0.01	0.01	0.29**	0.22**	−0.43**	1									
M3	0.10**	−0.04	0.04	−0.21**	−0.10**	−0.31**	−0.16**	1							
Character	−0.02	0.07**	−0.07**	0.31**	0.19**	−0.04	0.53**	−0.39**	1					
Competence	−0.06**	0.05	−0.04	0.30**	0.19**	−0.05*	0.54**	−0.43**	0.87**	1			
Confidence	−0.11**	0.04	−0.02	0.28**	0.17**	−0.05*	0.51**	−0.49**	0.77**	0.84**	1	
Connection	−0.09**	0.09**	−0.10**	0.33**	0.17**	−0.06*	0.52**	−0.47**	0.78**	0.80**	0.80**	1
*M*	–	–	–	9.53	8.33	3.09	3.37	2.83	3.98	3.83	3.65	3.68
*SD*	–	–	–	8.13	7.79	1.18	0.96	0.57	0.85	0.99	1.19	1.11

Gender and grade were dummy coded; gender: boys = 0, girls = 1; Grade 8: grade 7 = 0, grade 8 = 1; Grade 9: grade 7 = 0, grade 9 = 1.

X1 = natural environment contact, X2 = artificial environment contact, M1 = mindfulness, M2 = connectedness to nature, M3 = perceived stress.

*p < 0.05, **p < 0.01.

The dummy-coded variable for Grade 8 was positively and significantly correlated with natural environment contact, character, and connection (two subscales of PYD), suggesting that Grade 8 students scored higher in these variables compared to Grade 7 students. The dummy-coded variable for Grade 9 was negatively and significantly correlated with mindfulness, character, and connection, indicating that Grade 9 students scored lower in these variables compared to Grade 7 students.

Additionally, natural environment contact was positively and significantly correlated with artificial environment contact. Both natural and artificial environment contact were positively and significantly correlated with connectedness to nature and all four subscales of PYD, while they were negatively and significantly correlated with mindfulness and perceived stress.

### Impact of natural contact on positive youth development

Given the intercorrelations among natural environment contact, artificial environment contact, mindfulness, connectedness to nature, perceived stress, and the four subscales of PYD, a multiple mediation model was constructed using AMOS 26.0. In this model, mindfulness, connectedness to nature, and perceived stress were included as three parallel mediators. The results indicated that the model had a good fit: χ^2^/df = 5.68, GFI = 0.98, NFI = 0.98, TLI = 0.97, CFI = 0.98, RMSEA = 0.05.

After controlling for the effects of gender and grade, natural environment contact was found to positively and significantly predict adolescents’ PYD, whereas the predictive effect of artificial environment contact on PYD was not significant. Additionally, natural environment contact positively predicted connectedness to nature and negatively predicted perceived stress, but it did not have a significant predictive effect on mindfulness. In contrast, artificial environment contact negatively predicted mindfulness and positively predicted perceived stress, but it did not significantly predict connectedness to nature. Moreover, mindfulness and connectedness to nature positively predicted adolescents’ PYD, while perceived stress had a negative predictive effect on PYD.

**Figure 2:**
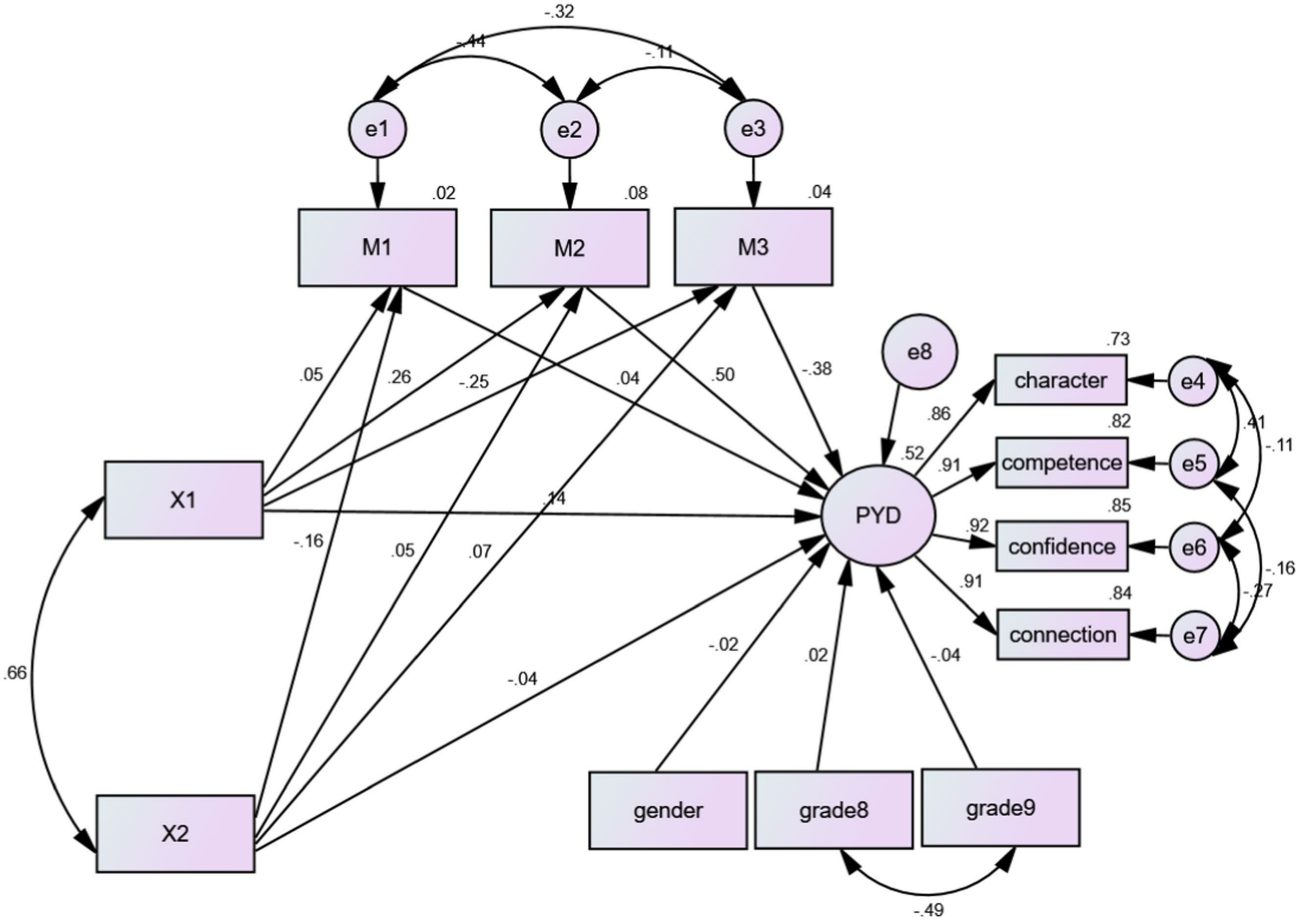
The multiple mediated model. Gender and grade were dummy coded; gender: boys = 0, girls = 1; Grade 8: grade 7 = 0, grade 8 = 1; Grade 9: grade 7 = 0, grade 9 = 1. X1 = natural environment contact, X2 = artificial environment contact, M1 = mindfulness, M2 = connectedness to nature, M3 = perceived stress. *p < 0.05, **p < 0.01.

These significant regression coefficients suggested that natural environment contact directly predicted adolescents’ PYD and also influenced PYD indirectly through its effects on connectedness to nature and perceived stress. In contrast, artificial environment contact did not directly affect adolescents’ PYD but predicted mindfulness negatively and significantly (see Table 2; Figure 2).

**Table 2:** The coefficient for multiple mediation model.

	Unstandardized estimate	S.E.	Standardized estimate	C.R.
PYD < gender	−0.03	0.03	−0.02	−1.16
PYD < grade 8	0.04	0.03	0.02	1.18
PYD < grade 9	−0.07	0.04	−0.04	−1.77
PYD < X1	0.01	0.00	0.14	5.80**
PYD < X2	0.00	0.00	−0.04	−1.78
M1 < X1	0.01	0.01	0.05	1.59
M1 < X2	−0.02	0.01	−0.16	−4.93**
M2 < X1	0.03	0.00	0.26	8.39**
M2 < X2	0.01	0.00	0.05	1.47
M3 < X1	−0.02	0.00	−0.25	−7.91**
M3 < X2	0.01	0.00	0.07	2.11*
PYD < M1	0.02	0.01	0.04	1.83
PYD < M2	0.38	0.02	0.50	21.75**
PYD < M3	−0.50	0.03	−0.39	−18.44**
character<PYD	1.00		0.86	
competence<PYD	1.23	0.02	0.91	57.23**
confidence<PYD	1.50	0.03	0.92	43.64
connection<PYD	1.39	0.04	0.91	40.14

Gender and grade were dummy coded; gender: boys = 0, girls = 1; Grade 8: grade 7 = 0, grade 8 = 1; Grade 9: grade 7 = 0, grade 9 = 1.

X1 = natural environment contact, X2 = artificial environment contact, M1 = mindfulness, M2 = connectedness to nature, M3 = perceived stress.

*p < 0.05, **p < 0.01.

Bootstrapping analysis with 5,000 resamples was conducted to calculate 95% bias-corrected confidence intervals (CI) for the effects. The results indicated that the total effect of natural environment contact on adolescents’ PYD was 0.37. The direct effect was 0.14, accounting for 37.84% of the total effect, while the indirect effect was 0.23, accounting for 62.16% of the total effect. The 95% CIs for the total, direct, and indirect effects did not contain zero, indicating that these effects were significant.

The mediating effects of connectedness to nature and perceived stress were 0.13 and 0.10, respectively, accounting for 56.52 and 43.48% of the total indirect effect; their 95% CIs also did not contain zero, indicating that both mediating effects were significant. However, the mediating effect of mindfulness was 0.002, and its 95% CI contained zero, suggesting that the mediating effect of mindfulness was not significant. Conversely, the 95% CIs for the total, direct, and indirect effects of artificial environment contact on adolescents’ PYD all contained zero, indicating that these effects were not significant (see Table 3).

**Table 3:** Mediated effect test with Bootstrap method.

		Effect	LLCI	ULCI	%
X1-PYD	Total effect	0.37	0.31	0.43	
	Direct effect	0.14	0.09	0.19	37.84% of total effect
	Indirect effect	0.23	0.18	0.27	62.16% of total effect
	The mediated effect of M1	0.002	−0.001	0.01	0.87% of indirect effect
	The mediated effect of M2	0.13	0.10	0.16	56.52% of indirect effect
	The mediated effect of M3	0.10	0.07	0.12	43.48% of indirect effect
X2-PYD	Total effect	−0.05	−0.11	0.01	
	Direct effect	−0.04	−0.08	0.002	
	Indirect effect	−0.01	−0.05	0.04	
	The mediated effect of M1	−0.01	−0.02	0.001	
	The mediated effect of M2	0.02	−0.01	0.05	
	The mediated effect of M3	−0.03	−0.05	0.003	

Gender and grade were dummy coded; gender: boys = 0, girls = 1; Grade 8: grade 7 = 0, grade 8 = 1; Grade 9: grade 7 = 0, grade 9 = 1.

X1 = natural environment contact, X2 = artificial environment contact, M1 = mindfulness, M2 = connectedness to nature, M3 = perceived stress.

## Discussion

This research aimed to examine the relationship between contact with nature and Chinese adolescents’ PYD and three parallel mediated effects (i.e., mindfulness, connectedness to nature, and perceived stress) on this relationship. With a questionnaire survey, the results indicated that compared to contact with artificial environments, natural environment contact not only directly contributed to adolescents’ PYD but also exerted an indirect effect through two pathways: connectedness to nature and perceived stress.

The findings demonstrated that exposure to natural environments had a direct positive effect on the development of Chinese adolescents. Specifically, the more adolescents contact with nature, the higher their level of positive development will be. In contrast, artificial environments do not provide the same positive effects. Scholar suggests that humans have evolved mechanisms for the unconscious recovery of positive emotions and stress relief in non-threatening natural environments over long periods of time (Ulrich, 1999). However, this mechanism does not adapt well to urban or built environments, where individuals must expend more cognitive resources to cope with or adapt to these settings, leading to cognitive resource overload and hindering development. Thus, Hypothesis 1 of this study is supported. These findings are also consistent with previous studies conducted with a western background (Bowers et al., 2021; Bates et al., 2018), underscoring the cross-cultural consistency of the beneficial effects of contact with nature on individuals and highlights the significant role of the natural environment as a developmental resource for adolescents.

In addition to the direct effect, nature contact also influenced adolescents’ positive development through two indirect pathways, with the indirect effects accounting for 62.16% of the total effect. The first pathway operated through connectedness to nature, which explained 56.52% of the total indirect effect, thus supporting Hypothesis 3. Frequent exposure to natural environments enhances adolescents’ sense of connection with nature, which, in turn, promotes their positive development. This is understandable. As the evolution has always been carried out in the nature, humans possess an innate tendency to be close to nature, a genetic trait shaped by evolution over millions of years (Wilson, 1986). In this case, people will be in a positive state when there are pro-survival natural environment cues (e.g., sparse forests and grasslands). For example, natural environments can stimulate the secretion of neurochemicals and hormones, affecting human’s immune system (Parsons et al., 1998). Engaging in outdoor activities and having contact with nature can help individuals develop the skills and confidence essential for survival (Bialeschki et al., 2007). This inherent predisposition has not diminished over time and continues to manifest in the need for humans, especially adolescents, to establish close contact with nature. However, the urban environment cues cannot trigger human positive reactions because of the relatively short urban-life history.

The second pathway was mediated through perceived stress, which accounted for 43.48% of the total indirect effect, supporting Hypothesis 4. Specifically, exposure to natural environments can reduce adolescents’ perceived stress, thereby promoting positive development. Previous research has shown that non-threatening landscape elements, greenery, and specific natural features require minimal cognitive resources to process and can help individuals recover from stress (Ulrich et al., 1991). Likewise, natural sounds (e.g., bird songs and flowing water) can reduce human skin conductivity and heart rate caused by stress (Parsons et al., 1998). Participants who visited the forest had stronger parasympathetic nervous activity, with lower levels of salivary cortisol, diastolic blood pressure and pulse rate than their peers who visit urban downtown (Park et al., 2009). It seems that the calming features of natural environments can restore the activation of the sympathetic nervous system caused by stress, leading to positive emotional experiences and ultimately fostering positive youth development.

However, the mediating effect of mindfulness was not found to be significant, indicating that Hypothesis 2 is not supported. This result contrasts with previous studies which have suggested that contact with nature helps restore attention (Taylor et al., 2002; Tennessen and Cimprich, 1995). One possible explanation for this discrepancy is that attention restoration theory emphasizes the unintentional aspect of nature contact, which facilitates the recovery of directed attention. In contrast, this study focused on the maintenance of intentional attention. Even though unintentional attention and intentional attention are often viewed as a trade-off. In reality, the maintenance of intentional attention does not necessarily require the use of unintentional attention, but rather than other mechanisms, such as activating positive emotions (Chen et al., 2016). Additionally, the design and content of the measurement tool used in this study could have influenced the results. The attention measured in this study differs from that measured in past studies, such as the functional perspective of attention assessed by Berman et al. (2008). Meanwhile, Kaplan and Kaplan (1989) summarized four characteristics of restorative environments: being away, fascination, extent, and compatibility. It is needed to examine whether the natural environment our participants contacted possesses these four characteristics.

Overall, the findings of this study support the Biophilia Hypothesis and the Stress Recovery Theory but do not provide evidence for the Attention Restoration Theory. Regarding the mediating effects, the Biophilia Hypothesis offers a stronger explanation for the positive impact of nature contact on the PYD of Chinese adolescents, followed by the Stress Recovery Theory. The difference in the explanatory power of these theories can be attributed to their distinct focuses. The Biophilia Hypothesis considers nature in a broad sense, encompassing the evolutionary environment of our ancestors, while the Attention Restoration Theory and the Stress Recovery Theory focus on specific natural elements in contemporary contexts (Chen et al., 2016). Moreover, the three theories emphasize different roles of nature: the Biophilia Hypothesis centers on nature’s role in survival adaptation, the Stress Recovery Theory emphasizes nature’s role in mental health, and the Attention Restoration Theory focuses on nature’s role in cognitive function. PYD is a broad concept characterized by traits such as character, competence, confidence, and connection, which are more closely linked to social adaptation and mental health. The first two theories — Biophilia and Stress Recovery — are more aligned with an individual’s intrinsic needs and instincts. The more adolescents experience contacts with nature, the closer they feel to it, effectively reducing stress and enhancing their level of positive development.

This study has several limitations. First, as a cross-sectional survey, it does not establish causality; therefore, causal relationships need to be further validated through longitudinal or experimental studies. Second, with convenience sampling method, the sample was drawn from Guangxi, a region in southern China known for its year-round greenery and beautiful natural scenery. Students in this region have more opportunities to interact with nature compared to those in less green environments. Whether these findings can be generalized to other populations, such as students in northern regions with less natural exposure, remains to be tested in future research. Third, the “contact with natural and artificial environment” instrument in the present study excluded blue elements of nature due to the Chinese school realities, which affect the ecological validity of the study. Besides, the study employed a more self-reported measure of attention, which might not fully capture the nuances of attentional restoration. Future research can further examine the different effect of the “blue” and the “green” elements of nature on individuals’ mental and behavioral outcomes.

However, it is worth noting that there are still some strengths for the present study. On the one hand, this study was employed the Chinese 4Cs model of PYD in a Chinese adolescent sample. The Chinese 4Cs model of PYD was constructed with Chinese traditional culture, and it can reflect the cultural and social realities in China and can represent the core of PYD in the new era of Chinese culture. To our knowledge, this is the first study which examine the relationship between contact with nature and the PYD of Chinese adolescents, and our study complements cross-cultural evidence of the benefits of nature contact. On the other hand, the study empirically tested three hypotheses related to nature contact—the Biophilia Hypothesis, the Stress Recovery Theory, and the Attention Restoration Theory—and demonstrated their varying explanatory power for PYD. It enriches and extends the existing evidence base by highlighting how nature contact can promote positive developmental outcomes, and provides more operational and practical implications to promote adolescent positive development. Finally, while most existing studies on nature exposure have focused on mitigating negative impacts on adolescents, this study approached the topic from the perspective of PYD, offering a more holistic view. It provides evidence that nature can serve as a valuable resource for promoting positive developmental outcomes. This perspective broadens the scope of current research and encourages future studies to adopt a more positive lens in examining the relationship between nature and individual development.

This study provides compelling evidence that nature contact is a vital resource for promoting PYD, which has significant implications for educators, parents, urban planners, and policymakers. Understanding the pathways through which natural environments contribute to well-being and personal growth can inform strategies to incorporate more natural elements into daily life, especially for young people in urbanized settings. By highlighting the mechanisms of connectedness to nature and stress reduction, this research underscores the importance of integrating natural spaces into school environments and communities to foster healthier developmental outcomes.

## Conclusion

The results of this investigation show that natural contact not only directly and positively predicts adolescents’ positive development, but also indirectly influences it through two pathways: connectedness to nature and perceived stress. These findings deepen our understanding of how nature contact leads to adolescents’ positive and healthful development, and provide empirical support for the Biophilia Hypothesis, and Stress Recovery Theory. It also has important implications for urban planning and school policies, suggesting that greater efforts are needed to ensure adolescents have more opportunities to engage with natural environments to promote their PYD. Noting that the survey focused on the green nature contact with convenience sampling method, further research in this field would be of great help in examining the effects of different elements of nature on individuals’ outcomes related to PYD with experimental methods and more representative samples.

## Data Availability

The original contributions presented in the study are included in the article/supplementary material, further inquiries can be directed to the corresponding author.
